# Implicit and Explicit Routes to Recognize the Own Body: Evidence from Brain Damaged Patients

**DOI:** 10.3389/fnhum.2016.00405

**Published:** 2016-08-31

**Authors:** Michela Candini, Marina Farinelli, Francesca Ferri, Stefano Avanzi, Daniela Cevolani, Vittorio Gallese, Georg Northoff, Francesca Frassinetti

**Affiliations:** ^1^Department of Psychology, University of BolognaBologna, Italy; ^2^Clinical Psychology Service, Villa Bellombra Rehabilitation HospitalBologna, Italy; ^3^Department of Psychology, University of EssexColchester, UK; ^4^IRCCS, Fondazione Salvatore MaugeriMantova, Italy; ^5^Neuroradiology Unit, Department of Neurosciences, Bellaria HospitalBologna, Italy; ^6^Department of Neuroscience, University of ParmaParma, Italy; ^7^School of Advanced Study, Institute of Philosophy, University of LondonLondon, UK; ^8^Institute of Mental Health Research, University of OttawaOttawa, Canada

**Keywords:** implicit and explicit dissociation, mental rotation, body-part, self-other recognition, brain damaged patient

## Abstract

Much research suggested that recognizing our own body-parts and attributing a body-part to our physical self-likely involve distinct processes. Accordingly, facilitation for self-body-parts was found when an implicit, but not an explicit, self-recognition was required. Here, we assess whether implicit and explicit bodily self-recognition is mediated by different cerebral networks and can be selectively impaired after brain lesion. To this aim, right- (RBD) and left- (LBD) brain damaged patients and age-matched controls were presented with rotated pictures of either self- or other-people hands. In the Implicit task participants were submitted to hand laterality judgments. In the Explicit task they had to judge whether the hand belonged, or not, to them. In the Implicit task, controls and LBD patients, but not RBD patients, showed an advantage for self-body stimuli. In the Explicit task a disadvantage emerged for self-compared to others' body stimuli in controls as well as in patients. Moreover, when we directly compared the performance of patients and controls, we found RBD, but not LBD, patients to be impaired in both the implicit and explicit recognition of self-body-part stimuli. Conversely, no differences were found for others' body-part stimuli. Crucially, 40% RBD patients showed a selective deficit for implicit processing of self-body-part stimuli, whereas 27% of them showed a selective deficit in the explicit recognition of their own body. Additionally, we provide anatomical evidence revealing the neural basis of this dissociation. Based on both behavioral and anatomical data, we suggest that different areas of the right hemisphere underpin implicit and explicit self-body knowledge.

## Introduction

The body, including its various parts, is an important component of our self and its identity, as well as one of its most distinctive physical features. Previous studies showed that the recognition of a body (or body-parts) as one's own depends on a multitude of information. These studies suggest that bodily self-recognition results from the simultaneous processing of visual components (Sugiura et al., 2005; Devue et al., 2007), somatosensory and proprioceptive signals (for a review see Blanke, 2012), and motor information (Sugiura et al., 2006; Frassinetti et al., 2009). Starting from this evidence here we focus on the contribution of visual and motor information to bodily self-processing. A relevant distinction has been recently made in the field between implicit and explicit body knowledge. In this respect, Frassinetti et al. (2008, 2009, 2010) investigated the implicit recognition of self-body-parts by using a visual matching-to-sample task. Participants were required to decide which of two vertically aligned images (high or low) matched the central target stimulus (i.e., an Implicit task). Stimuli could depict participants' or other people's body-parts (hand, foot, arm, leg). Results showed that participants were more accurate with self rather than others' body-parts. This facilitation was called *self-advantage effect*. Interestingly, the self-advantage effect was not found when participants were explicitly required to judge whether the upper or the lower stimulus corresponded to their own body-parts (Frassinetti et al., 2011). This suggests possible dissociation between implicit and explicit bodily self-processing. However, neither such implicit-explicit dissociation in the self-advantage effect nor its underlying neural correlates have been demonstrated so far in brain damaged patients.

To better investigate the mechanisms of the implicit and explicit bodily self-processing, in a following study the authors adopted a laterality judgment task (Ferri et al., 2011). In a first experiment (implicit), participants were requested to report the laterality of images depicting self or other's hands presented at different angular orientations, whereas in the second experiment (explicit), participants were asked to recognize their own hand (Ferri et al., 2011). In order to perform the former but not the latter task, participants simulated a motor rotation of their own body-parts so as to match that of the observed stimulus (Ionta et al., 2007, 2012). In the laterality judgment task, a facilitatory effect (i.e., faster response times) was found in response to hand stimuli belonging to the participants (self-stimuli), suggesting that the body self-advantage is based on a sensorimotor representation. This facilitatory effect was not observed in the second task, that is, during the explicit discrimination between self and others' stimuli. Indeed, participants performed worst with self-compared to others' stimuli. The authors hypothesize that to successfully recognize a stimulus as own body-part, participants compare the displayed picture with the mental representation of one's own body, using visual cue and information arise from memory. However, this representation may be affected by perceptual distortions, such as an overestimation of the body size or distorted body shape. Thus, when participants match the image of their own body-part with the displayed hand, the judgment of ownership is more vulnerable to errors than the implicit one. Overall, these results raise the possibility that bodily self-recognition is based on, at least, two different mechanisms for the implicit and explicit self-body processing, subtended by two different cerebral networks. As a consequence, different brain lesions might selectively impair either the implicit or the explicit self-body processing.

To test these hypotheses, patients with focal cerebral lesion (15 RBD and 15 LBD patients) and a group of healthy subjects were recruited and asked to perform two experiments.

In the first experiment participants were submitted to a laterality judgment task of rotated hands with different angular orientation (Implicit task). In the second experiment they were asked to explicitly recognize their own hand (Explicit task; see Ferri et al., 2011 for the experimental paradigms). In both experiments, the displayed hand was the participants' hand (self-condition) in half of the trials, whereas it depicted other people's hand (other condition) in the rest of the trials.

We expected to find one of the following alternative outcomes. If implicit and explicit body processing are mediated by different neural networks, then at least some of the patients showing poor performance in the Implicit task should perform similarly to controls in the Explicit task, or the opposite. This does not hold, indeed, in cases where the lesion includes brain regions involved in both tasks. If, in contrast, implicit and explicit self-body-parts recognition is mediated by the same network, all patients should perform worse than controls in both the implicit and the explicit tasks.

## Materials and methods

### Participants and neuropsychological assessment

Fifteen RBD patients (9 males, age = 59.3.4 ± 7 years; education = 10.9 ± 4.7 years) and 15 LBD patients (10 males, age = 63.1 ± 7 years; education = 8.9 ± 2.7 years) participated in the study. All patients were right handed by their own verbal report and were assessed for the presence of a general cognitive impairment through the Mini-Mental State Examination (Folstein et al., 1975). Thirty healthy volunteers were recruited through a recreational center as controls: half of them were matched with the RBD patients, whereas the other half were matched with the LBD patients. Three one-way ANOVAs confirmed that the four groups were not significantly different for age [*F*_(3, 60)_ = 4.46, *p* = 0.13], education [*F*_(3, 60)_ = 5.63, *p* = 0.17] and MMSE score [*F*_(3, 54)_ = 2.07, *p* = 0.17]. Finally, no significant difference was found for the variable sex across the four groups, as a chi-square test confirmed [$\chi_{\left(1\right)}^{2}$ = 1.07, *p* = 0.30].

The presence and severity of extrapersonal neglect (Bell's Cancellation test; Gauthier et al., 1989), personal neglect (Fluff Test; Cocchini et al., 2001) and anosognosia for hemiplegia and hemianestesia (Spinazzola et al., 2008) were also assessed (for details see Table 1).

**Table 1:** **Clinical and neuropsychological data of right brain damaged (a) and left brain damaged patients (b)**.

**A**								
**Patient**	**AGE**	**TPL**	**AETIOLOGY**	MMSE^*^	Bells^**^	**Fluff**	AHP^***^	AHE^***^
RBD 1	79	60	I	30	**5**	2	0	0
RBD 2	73	39	I	30	0	0	0	0
RBD 3	48	19	I	–	**5**	1	0	0
RBD 4	39	228	I	–	2	1	0	0
RBD 5	57	650	I	–	3	0	0	0
RBD 6	59	50	I	–	0	0	0	0
RBD 7	62	30	I	28	0	0	0	0
RBD 8	54	392	H	28	2	1	0	0
RBD 9	71	39	I	22	**15**	0	0	0
RBD 10	65	73	H	24	**14**	**10**	**1**	**1**
RBD 11	61	37	I	–	0	0	0	**1**
RBD 12	64	79	H	23	**12**	**5**	0	**2**
RBD 13	68	16	I	28	0	1	0	0
RBD 14	34	50	H	30	0	1	0	0
RBD 15	55	96	I	30	3	0	0	0
**B**								
**Patient**	**AGE**	**TPL**	**AETIOLOGY**	MMSE^*^	**Token Test**			
LBD 1	65	70	H	–	–			
LBD 2	44	93	H	27	26			
LBD 3	57	25	I	22	33			
LBD 4	77	43	I	28	–			
LBD 5	67	52	I	22	26			
LBD 6	51	34	H	27	32			
LBD 7	47	51	I	24	30			
LBD 8	61	47	H	30	34			
LBD 9	52	35	H	25	30			
LBD 10	63	31	I	28	32			
LBD 11	64	52	I	28	31			
LBD 12	72	28	I	20	22			
LBD 13	75	39	I	26	34			
LBD 14	82	60	I	25	30			
LBD 15	70	95	I	28	32			

*TPL, Time post lesion (days); I, ischemic stroke, H, hemorrhagic stroke*;

* *MMSE, Mini Mental State Examination (scores are corrected for years of education and age)*;

** *Bells Test, left omissions; Fluff test, omissions*;

*** *AHP, anosognosia for hemiplegia*;

*** AHE, anosognosia for hemianestesia (scoring 0 = no anosognosia, 1 = moderate anosognosia, 2 = severe anosognosia, each value refers to the left upper limb). Bold characters indicated pathological performance.

Patients were recruited at the Fondazione Maugeri Hospital (Castel Goffredo, Italy) and at the Villa Bellombra Rehabilitation Hospital (Bologna, Italy).

All participants, naive to the purpose of the study, gave their informed consent to participate to the study. The study was approved by the local ethics committee (Villa Bellombra Hospital and Department of Psychology of Bologna), and all procedures were in agreement with the 2008 Helsinki Declaration.

### Patients' lesion

Brain lesions of 12 RBD and 12 LBD were identified by Computerized Tomography and Magnetic Resonance digitalized images (CT/MRI). For each patient, the location and extent of brain damage was delineated and manually mapped in the stereotactic space of the MNI by using the free software MRIcro (Rorden and Brett, 2000).

As first step, MNI template was rotated (pitch only) to approximate the slice plane of the patient's scan. A trained rater (MC), using anatomically landmarks, manually mapped the lesion onto each correspondent template slice. After that, drawn lesions were inspected by a second trained rater (FF) and in case of disagreement, an intersection lesion map was used. Finally, lesions maps were rotated back into the standard space applying the inverse of the transformation parameters used on the stage of adaptation to the brain scan.

To compare lesions' extension we conducted a Mann Whitney *U*-test on the mean number of voxels involved by the lesion for each patients in the RBD patients' group and LBD patients' group. Results confirmed that the two groups were not significantly different regarding the ‘total lesion volume’ [*U* = 47.00; *z* = −1.68; *p* = 0.09].

**Figure 1:**
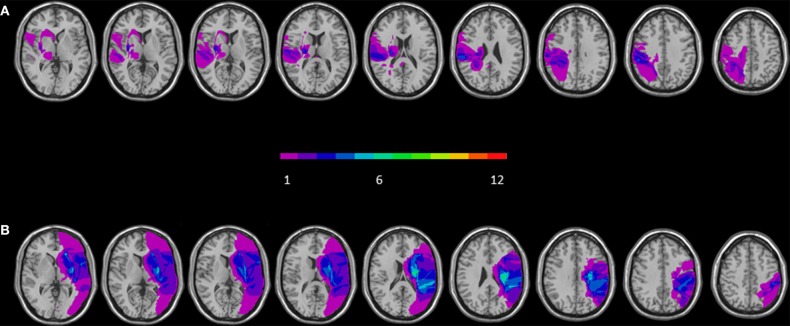
**Overlay of reconstructed lesion plots of LBD (A) and RBD patients (B) superimposed onto MNI template**. The number of overlapping lesions is illustrated by different colors coding from violet (*n* = 1) to green (*n* = 7).

The maximum lesion overlap of RBD patients' lesions was mainly located along two different regions: one encompassing frontal subcortical region (putamen, paraventricular area, internal and external capsule) and one involving temporo-parietal regions such as the insular cortex, the superior temporal and postcentral gyri and the inferior parietal lobe (BA 40) (for a graphical representation, see Figure 1A). The maximum lesion overlap of LBD patients' lesions involved a frontal subcortical region (paraventricular area, internal and external capsule), the postcentral gyrus and the inferior parietal lobe (BA 40) (for a graphical representation, see Figure 1B).

### Behavioral studies

#### Stimuli and procedure

**Figure 2:**
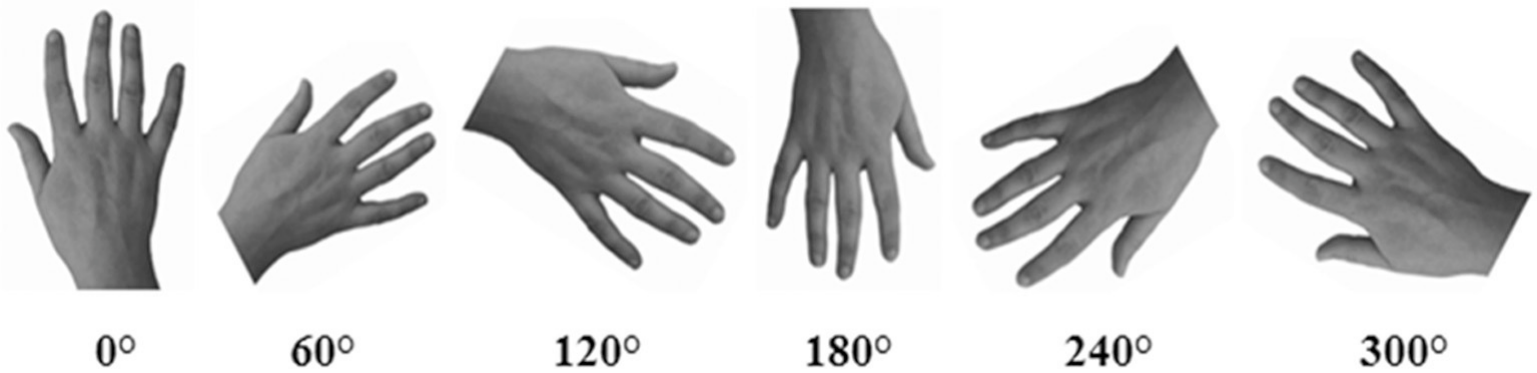
**An example of stimuli representing hands at different orientations**. In the Implicit task, participants were required to judge the laterality of each stimulus. In the Explicit task, participants were required to judge if the hand was or was not their own.

Gray-scale pictures of the dorsal view of right and left hands (see Figure 2) were used as experimental stimuli. We adopted only the dorsal view of hand to compare the present findings with the previous ones of our group (Ferri et al., 2011). The hands of each participant were photographed with a digital camera in a session prior to the experiments. Hands were always photographed with constant artificial light, in the same position and at a fixed distance from the camera (40 cm).

Pictures were modified with Adobe Photoshop® CS4 software: each hand was cut from the original picture, centered and then pasted on a white background. Finally, each photograph was clockwise rotated to obtain six predefined orientation (0°, 60°, 120°, 180°, 240°, 300°), in which fingers pointing upwards defined the upright orientation. Half of the trials (*n* = 144) depicted the participant's own left or right hand (“self” trials), whereas the other half depicted the right or left hand of three other people (“other” trials). As far as the latter one, three stimuli were selected from a database of hands pictures as the best match with each participant's hand for size, age, skin color and gender. The luminosity of the gray-scale picture was adjusted taking into account the individually skin shades.

Participants sat in front of a PC screen, at a viewing distance of about 40 cm. A central fixation cross (500 ms duration) was presented at the beginning of each trial followed by a display containing hand's picture on a white background. Stimuli presentation was controlled by E-Prime 2.0 (Psychology Software Tools Inc.) and each trial was timed-out by the participant's response (up to 4000 ms).

In Experiment 1 participants had to judge the laterality (left or right) of displayed hand by pressing as accurately as possible and within the allowed time interval, a left or a right response key (“R” or “P” on keyboard). In Experiment 2, participants had to explicitly judge whether the displayed hand corresponded or not to their own hand by pressing as accurately as possible and within the allowed time interval, a left or a right previously assigned response key (“R” or “P” on keyboard). In both Experiments the response keys were counterbalanced between subjects. Since patients responded by using their not affected hand, that's the left for RBD and the right for LBD, healthy subjects were accordingly divided in two groups: 15 who responded by using the index finger of the right hand, and 15 who using their left hand.

Patients with neglect and/or left hemianopia were submitted to an adapted version of Implicit and Explicit Task in which all stimuli were displayed on the right side of the screen. Analogously, an adapted version of both experiments to patients showing right hemianopia was designed by shifting all stimuli to the left side of the screen.

Both experiments were always preceded by 8 trials as practice. Then, each experiment comprised 288 trials, 72 trials for each of the four conditions: self-right hand, self-left hand, other-right hand, and other-left hand. Furthermore, each orientation was randomly presented 12 times per condition. Since Experiment 1 investigated the implicit and Experiment 2 the explicit bodily self-recognition, Experiment 1 was always conducted before Experiment 2. All participants performed both experiments in one single session lasting up to 1 h.

#### Statistical analyses

Data from Experiment 1 (Implicit task) and Experiment 2 (Explicit task) were analyzed separately on mean response times (RTs) for correct trials and on the percentage of correct responses (accuracy). First of all, the presence of the self-advantage effect and the strategy to solve the task (i.e., the mental rotation) were separately tested in healthy subjects and in RBD and LBD patients (Analysis on each group). For healthy subjects, an ANOVA was conducted with Owner (self and other), Laterality (left and right displayed hand) and Orientation (0°, 60°, 120°, 180°, 240°, 300°) as within-subjects factors and Group (H-R = healthy subjects responding with the right finger and H-L = healthy subjects responding with the left finger) as between-subjects factor. For patients, separate ANOVAs were conducted for RBD and LBD patients with Owner (self and other), Laterality (left and right displayed hand) and Orientation (0°, 60°, 120°, 180°, 240°, 300°) as within-subjects factors.

Subsequently, we directly compared patients' performance with the healthy subjects group using the same hand in performing the task. For this reason, separate ANOVAs were conducted (Patients and healthy subjects comparison), with Owner, Laterality, as within-subjects factors: the first, between RBD patients and healthy subjects responding with the right finger (H-R) and the second, between LBD patients and healthy subjects responding with the left finger (H-L). Since these analyses were conducted to compare patients' and controls' performance, only the variable Group and its interaction with other variables will be reported.

Finally, we compared the performance of the four groups on a self-advantage index (i.e., self-minus other). Accordingly, we conducted two One-Way ANOVAs on RTs and on percentage of correct responses, separately for Implicit and Explicit task considering the Group factor (H-R, H-L, LBD, and RBD patients). Where necessary, post-hoc analyses were conducted by using Bonferroni's correction. The magnitude of effect size was expressed by η^2^_*p*_.

## Results

### Within-group results of experiment 1 (implicit task)

#### Analysis on healthy subjects

**Figure 3:**
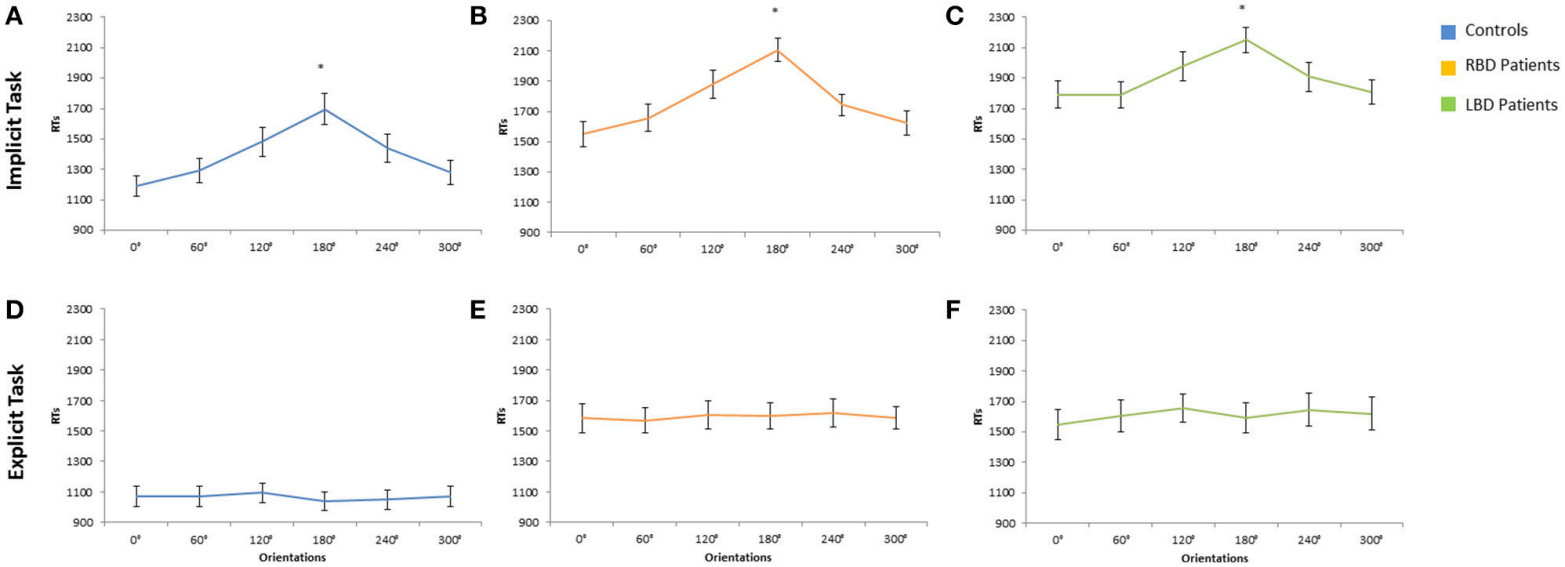
**Mean response times of controls, right brain damaged (RBD) and left brain damaged (LBD) patients in Implicit (A–C) and Explicit (D–F) task, respectively**. Results are displayed as a function of displayed hand Orientation. The significant difference between 180° and all other orientations is starred. Error bars depict SEMs. The “^*^”indicate the significant difference.

The main effect of **Owner** was significant [*F*_(1, 28)_ = 6.14; *p* < 0.02; η^2^_*p*_ = 0.18]: participants responded faster to self than to other people's hand (self = 1370 ms vs. other = 1421 ms). The main effect of **Orientation** was significant [*F*_(5, 140)_ = 54.74 *p* < 0.0001; $\eta_{p}^{2}$ = 0.66], since RTs to stimuli at 180° (1696 ms) were longer than all other orientations (0° = 1189 ms, 60° = 1291 ms, 120° = 1481 ms, 240° = 1438 ms, 300° = 1279 ms, *p* < 0.0001 in all cases; see Figure 3A). Moreover, longer RTs were observed at 120° and 240° compared to RTs at 0°, 60°, and 300°, *p* < 0.005 for all comparisons). These results show that participants used mental rotation strategy to solve the Implicit task, both for right and left stimuli.

The variable Group and its interaction with other variables were not significant suggesting that the responding hand did not influence the described effects.

**Figure 4:**
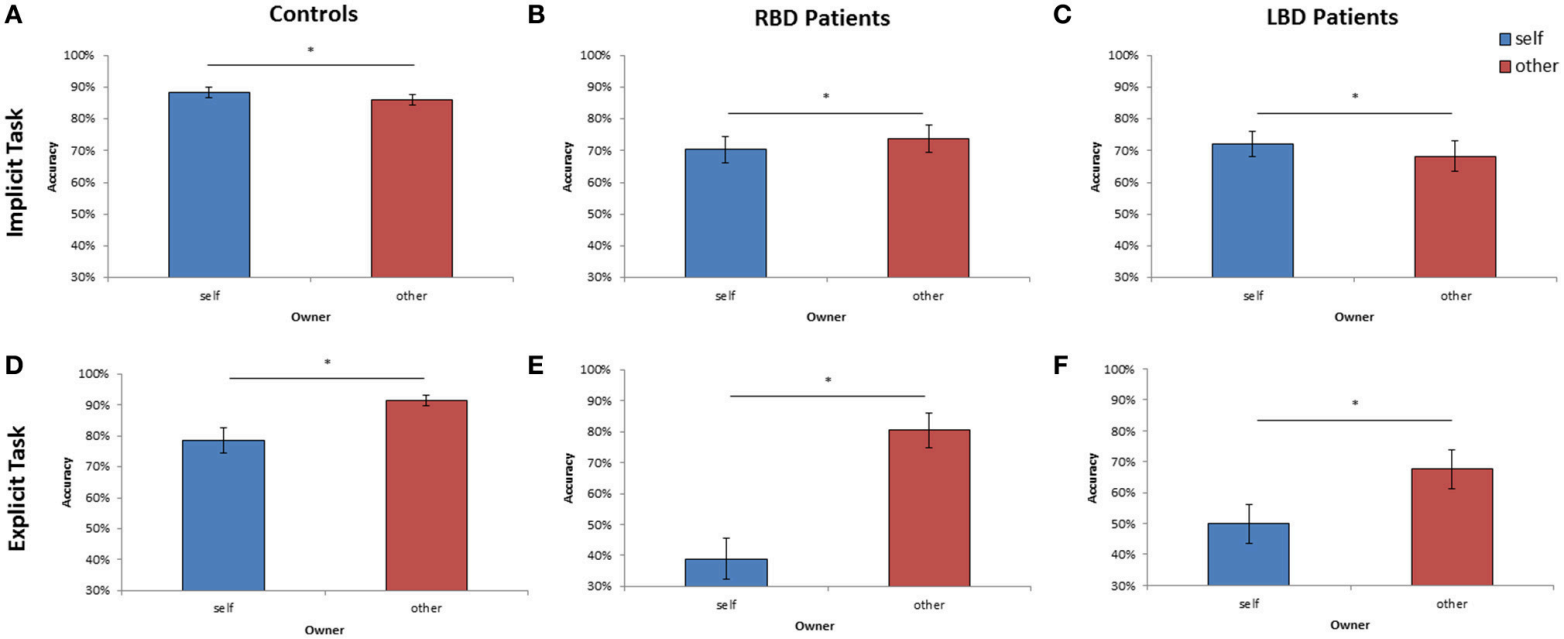
**Mean of correct response of controls, right brain damaged (RBD) and left brain damaged (LBD) patients in Implicit (A–C) and Explicit (D–F) task, respectively**. The significant difference between self and other stimuli is starred. Error bars depict SEMs. The “^*^”indicate the significant difference.

As far as the percentage of correct responses, similar results were found: the main effect of **Owner** was significant [*F*_(1, 28)_ = 11.95; *p* < 0.002; $\eta_{p}^{2}$ = 0.30]: participants were more accurate with self than with other people's hand (self = 88% vs. other = 86%; see Figure 4A). The main effect of **Orientation** was significant [*F*_(5, 140)_ = 20.72; *p* < 0.0001; $\eta_{p}^{2}$ = 0.43], since participants were less accurate at 180° (73%) than all other orientations (0° = 93%, 60° = 93%, 120° = 86%, 240° = 86%, 300° = 92%, *p* < 0.03 in all cases). Crucially, the interaction **Owner** × **Laterality** was significant [*F*_(1, 28)_ = 4.40; *p* < 0.05; $\eta_{p}^{2}$ = 0.14]: when the right hand is displayed, participants were more accurate with self (89%) compared to other people's hand (85%; *p* < 0.01). No significant difference was found for the left hand instead. The variable Group and its interaction with other variables were not significant suggesting that the responding hand did not influence the described effects.

#### RBD patients

The variable **Orientation** was significant [*F*_(1, 14)_ = 50.22; *p* < 0.0001; $\eta_{p}^{2}$ = 0.78]: since RTs to stimuli at 180° (2107 ms) were longer than all other orientations (0° = 1551 ms, 60° = 1657 ms, 120° = 2107 ms, 240° = 1742 ms, 300° = 1623 ms, *p* < 0.001 in all cases; see Figure 3B).

The interaction **Laterality** × **Orientation** was significant [*F*_(5, 70)_ = 5.72; *p* < 0.001; $\eta_{p}^{2}$ = 0.29]: participants responded faster when the right (ipsilesional) hand is rotated at 0° (1428 ms) compared to the left (contralesional hand) hand (1674 ms) but these variables did not interact with Owner.

Analysis on the percentage of correct responses, put in evidence a significant effect of **Owner** [*F*_(1, 14)_ = 4.59; *p* < 0.05; $\eta_{p}^{2}$ = 0.25] since RBD patients were *less* accurate with self than with other people's hand (self = 70% vs. other = 74%; see Figure 4B). Furthermore, the variable **Orientation** was significant [*F*_(1, 14)_ = 12.95; *p* < 0.0001; $\eta_{p}^{2}$ = 0.48]: participants were less accurate at 180° (52%) than all other orientations (0° = 81%, 60° = 77%, 120° = 67%, 240° = 76%, 300° = 80%, *p* < 0.03 in all cases).

#### LBD patients

The main effect of **Owner** was significant [*F*_(1, 14)_ = 7.57; *p* < 0.02; $\eta_{p}^{2}$ = 0.35]: participants responded faster with self than with other people's hand (self = 1869 ms vs. other = 1938 ms).

The variable **Orientation** was significant [*F*_(1, 14)_ = 9.77; *p* < 0.0001; $\eta_{p}^{2}$ = 0.41]: since RTs to stimuli at 180° (2150 ms) were longer than all other orientations (0° = 1791 ms, 60° = 1790 ms, 120° = 1977 ms, 240° = 1907 ms, 300° = 1808 ms, *p* < 0.05 in all cases; see Figure 3C).

Analysis on the percentage of correct responses, confirmed a significant effect of **Owner** [*F*_(1, 14)_ = 4.41; *p* < 0.05; $\eta_{p}^{2}$ = 0.24], being the LBD patients more accurate with self than with other people's hand (self = 72% vs. other = 68%; see Figure 4C). Furthermore, the variable **Orientation** was significant [*F*_(1, 14)_ = 7.73; *p* < 0.0001; $\eta_{p}^{2}$ = 0.35]: since participants were less accurate at 180° (58%) than all other orientations (0° = 75%, 60° = 75%, 120° = 69%, 240° = 71%, 300° = 74%, *p* < 0.02 in all cases).

### Within-group results of experiment 2 (explicit task)

#### Analysis on healthy subjects

The main effect of **Owner** was significant [*F*_(1, 28)_ = 13.62; *p* < 0.001; $\eta_{p}^{2}$ = 0.32]: RTs were longer for self (1200 ms) than for other people's stimuli (930 ms) showing the so called self-disadvantage effect.

The variable Orientation was not significant [*F*_(5, 140)_ = 1.20; *p* > 0.05], suggesting that participants did not use mental rotation strategy to solve the Explicit task (0° = 1068 ms, 60° = 1069 ms, 120° = 1092 ms, 180° = 1040 ms, 240° = 1050 ms, 300° = 1070 ms; see Figure 3D).

As far as the percentage of correct responses, the following results were found: the main effect of **Owner** was significant [*F*_(1, 28)_ = 12.09; *p* < 0.001; $\eta_{p}^{2}$ = 0.30]: healthy participants were *less* accurate with self (79%) than with others' stimuli (91%; see Figure 4D) showing the so called self-disadvantage effect.

The variable Orientation was not significant [*F*_(5, 140)_ = 0.92; *p* = 0.47], suggesting that participants did not use mental rotation strategy to solve the Explicit task (0° = 83%, 60° = 87%, 120° = 85%, 180° = 85%, 240° = 85%, 300° = 85%).

#### RBD patients

The main effect of **Owner** was significant [*F*_(1, 14)_ = 37.28; *p* < 0.001; $\eta_{p}^{2}$ = 0.73]: RTs were longer for self (1975 ms) than for other people's stimuli (1212 ms) showing the so called self-disadvantage effect.

The variable Orientation was not significant [*F*_(5, 140)_ = 1.20; *p* > 0.05], suggesting that RBD patients did not use mental rotation strategy to solve the Explicit task (0° = 1583 ms, 60° = 1569 ms, 120° = 1607 ms, 180° = 1600 ms, 240° = 1617 ms, 300° = 1586 ms, see Figure 3E).

Concerning the percentage of correct responses, RBD patients were *less* accurate with self than with other people's hand [39% vs. 80%, *F*_(1, 14)_ = 20.47; *p* < 0.005; $\eta_{p}^{2}$ = 0.44; see Figure 4E] and were *less* accurate with left than with right hand [54% vs. 65%, *F*_(1, 14)_ = 10.78; *p* < 0.0001; $\eta_{p}^{2}$ = 0.59]. The interaction **Owner** × **Laterality** was significant [*F*_(1, 14)_ = 8.17; *p* < 0.01; $\eta_{p}^{2}$ = 0.37]: when the displayed stimulus belonged to participants, they were less accurate with the left contralesional hand (29%) compared to the right ipsilesional responding hand (49%; *p* < 0.003), conversely this effect was not found with others' stimuli (left = 79% vs. right = 81%; *p* = 0.99).

#### LBD patients

The main effect of **Owner** was significant [*F*_(1, 14)_ = 17.81; *p* < 0.001; $\eta_{p}^{2}$ = 0.60]: RTs were longer for self (1740 ms) than for other people's stimuli (1483 ms) showing the so called self-disadvantage effect.

The variable Orientation was not significant [*F*_(5, 140)_ = 1.20; *p* > 0.05], suggesting that LBD patients did not use mental rotation strategy to solve the Explicit task (0° = 1549 ms, 60° = 1605 ms, 120° = 1656 ms, 180° = 1593 ms, 240° = 1645 ms, 300° = 1619 ms, see Figure 3F).

Again, analysis on the percentage of correct responses, put in evidence that LBD patients were *less* accurate with self than with other people's hand [50% vs. 68%, *F*_(1, 14)_ = 12.89; *p* < 0.003; $\eta_{p}^{2}$ = 0.48, see Figure 4F].

### Between-group results of experiment 1 (implicit task)

#### RBD patients and healthy subjects responding with the right finger (H-R)

The variable **Group** was significant [*F*_(1, 28)_ = 9.66; *p* < 0.004; $\eta_{p}^{2}$ = 0.26], which was mainly due to longer response time in RBD patients (1760 ms) compared to controls (1358 ms).

The interaction **Owner** × **Group** was significant [*F*_(1, 28)_ = 5.89, *p* < 0.02; $\eta_{p}^{2}$ = 0.17]: RBD patients responded slower than controls when the displayed hand belonged to them (1804 ms vs. 1326 ms; *p* < 0.007) but not with other people's hand (1716 ms vs. 1390 ms; *p* = 0.12).

**Figure 5:**
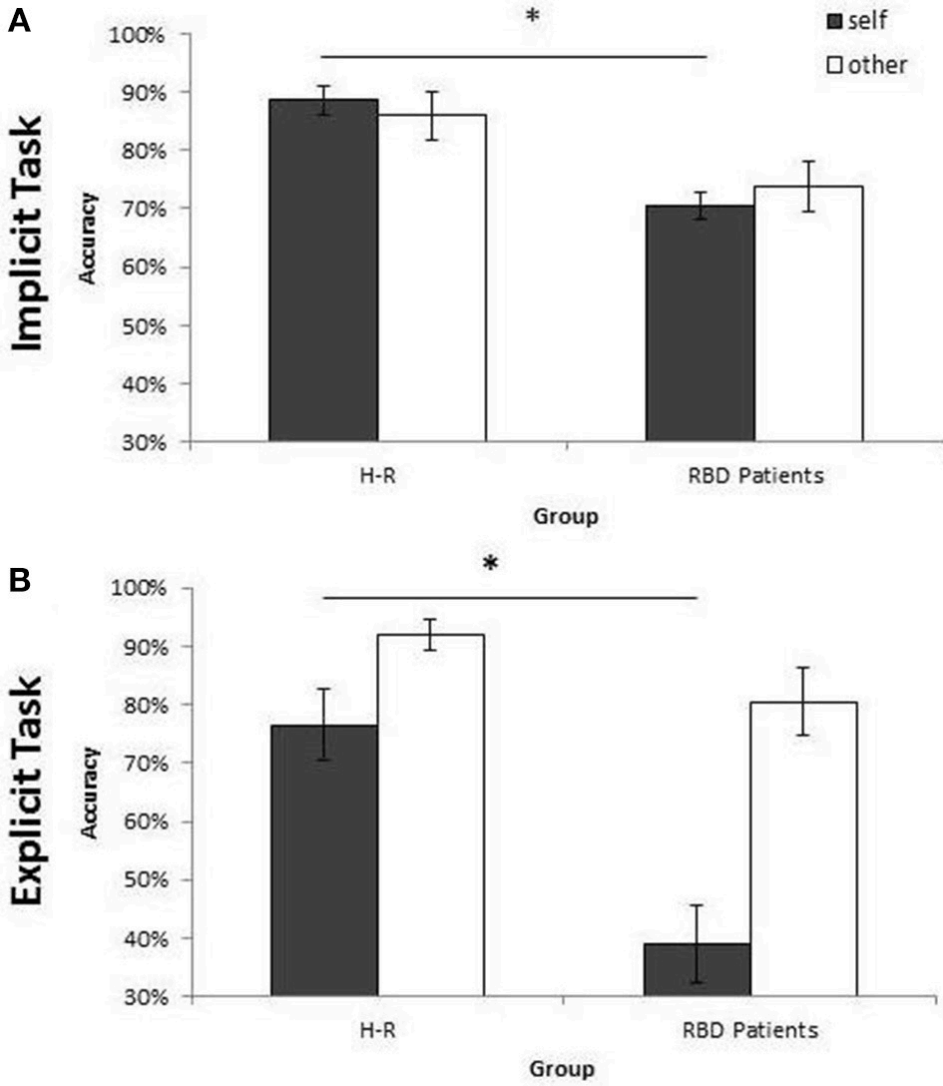
**Mean of correct response of Controls and RBD in the Implicit (A) and Explicit (B) task**. Results are displayed as a function of ownership (self/other). Between-group significant difference are starred. Error bars depict SEMs. The “^*^”indicate the significant difference.

Similar results were obtained when the percentage of correct responses were analyzed: RBD patients were less accurate than controls [72% vs. 87%; *F*_(1, 28)_ = 10.37; *p* < 0.003; $\eta_{p}^{2}$ = 0.27]. Moreover, the interaction **Owner** × **Group** [*F*_(1, 28)_ = 11.33; *p* < 0.002; $\eta_{p}^{2}$ = 0.29] showed a selective deficit of RBD patients compared to controls with self (70% vs. 89%; *p* < 0.004) but not with other people's stimuli (74% vs. 86%; *p* = 0.10; see Figure 5A).

#### LBD patients and healthy subjects responding with the left finger (H-L)

The variable **Group** was significant considering both RTs, since LBD patients were slower than controls [1904 ms vs. 1433 ms; *F*_(1, 28)_ = 9.12; *p* < 0.005; $\eta_{p}^{2}$ = 0.25] and accuracy, because LBD patients were less accurate than controls [70% vs. 87%; *F*_(1, 28)_ = 12.34, *p* < 0.001; $\eta_{p}^{2}$ = 0.31]. The interaction Owner × Group was not significant, neither for RTs [*F*_(1, 28)_ = 0.62, *p* = 0.44] nor for accuracy [*F*_(1, 28)_ = 0.83, *p* = 0.37].

#### RBD, LBD patients, and healthy subjects (H-L and H-R)

The ANOVA conducted on the self-advantage index (self-minus other) showed a significant effect of the variable **Group** considering both RTs [*F*_(3, 56)_ = 3.92; *p* < 0.01; η^2^_p_ = 0.17] and accuracy [*F*_(3, 56)_ = 3.10; *p* < 0.03; η^2^_p_ = 0.14], since RBD patients performed worse compared to three groups (all ps < 0.005).

### Between-group results of experiment 2 (explicit task)

#### RBD patients and healthy subjects responding with the right finger (H-R)

The variable **Group** was significant [*F*_(1, 28)_ = 12.17, *p* < 0.001; η^2^_p_ = 0.30]: RBD patients responded slower (1594 ms) than controls (1119 ms). The interaction **Owner** × **Group** was significant [*F*_(1, 28)_ = 5.92; *p* < 0.02; $\eta_{p}^{2}$ = 0.17] since with self-stimuli, RBD patients performed worse than controls (1293 ms; *p* < 0.001) whereas no such effect was found with other people's stimuli (945 ms; *p* = 0.61).

Analysis conducted on the percentage of correct responses put in evidence a similar pattern of results. Again, RBD patients were less accurate than controls [60% vs. 84%; *F*_(1, 28)_ = 18.74; *p* < 0.001; $\eta_{p}^{2}$ = 0.40]. The interaction **Owner** × **Group** [*F*_(1, 28)_ = 5.94; *p* < 0.02; $\eta_{p}^{2}$ = 0.17] confirmed that the impairment of RBD patients compared with controls was selective for self-hand (39% vs. 76%; *p* < 0.001) and not for other people's hand (80% vs. 92%; *p* = 0.89; see Figure 5B).

#### LBD patients and healthy subjects responding with the left finger (H-L)

The variable **Group** was significant both for RTs, being LBD patients slower than controls [1611 ms vs. 1010 ms; *F*_(1, 28)_ = 24.8; *p* < 0.0001; $\eta_{p}^{2}$ = 0.47] and accuracy, since LBD patients were less accurate than controls [59% vs. 86%; *F*_(1, 28)_ = 14.97; *p* < 0.001; $\eta_{p}^{2}$ = 0.35]. The interaction **Owner** × **Group** was not significant, neither for RTs [*F*_(1, 28)_ = 0.37, *p* = 0.55] nor for accuracy [*F*_(1, 28)_ = 1.07, *p* = 0.31].

#### RBD, LBD patients, and healthy subjects (H-L and H-R)

The ANOVA conducted on the self-advantage index (self-minus other) showed a significant effect of the variable **Group** considering both RTs [*F*_(3, 56)_ = 6.45; *p* < 0.01; $\eta_{p}^{2}$ = 0.26] and accuracy [*F*_(3, 56)_ = 4.54; *p* < 0.001; $\eta_{p}^{2}$ = 0.26], since RBD patients performed worse compared to three groups (all *p*s < 0.007).

## Dissociations between implicit and explicit self-body knowledge

To sum up, previous analysis on RTs and accuracy showed that all participants adopted the mental rotation strategy to solve the laterality task (Implicit task), but not to perform the owner recognition task (Explicit task). Furthermore, in the Implicit Task a self-advantage emerged in controls and LBD patients, whereas a lack of this facilitation was found in RBD patients. Specifically, RBD patients were selectively impaired compared to controls in implicit processing self-body-parts. In the Explicit task, a self-disadvantage emerged in all groups of participants, and again RBD patients were selectively impaired compared to controls in self-body-parts processing.

Thus, in line with the aim of the present study, it is crucial to investigate possible dissociation in the implicit or in the explicit processing of self-body-parts in RBD patients and its neural correlates.

### Behavioral data on implicit and explicit dissociation in RBD patients

Since in the group analysis possible dissociations may have gone unobserved because of the well-known averaging artifact (Shallice, 1988), we compared the performance of each patient with the performance of the control group for self body-parts (mean percentage of accuracy obtained for self-trials collapsing the variables Orientation and Laterality), separately in the Implicit and in the Explicit task, by using a modified *t*-test which takes into account the size of the control group (Crawford et al., 2010). This comparison revealed that 10 out of 15 RBD patients were selectively impaired in the implicit or in the explicit self-body processing compared to controls: 6/15 (40%) were selectively impaired in the implicit and 4/15 (27%) in the explicit self-body processing (for statistical details see Supplemental Material).

### Lesion study on implicit and explicit dissociation in RBD patients

Since two distinct deficits were observed within the RBD patients group, we separately overlaid lesions of patients with impaired implicit and explicit self-body-parts recognition.

**Figure 6:**
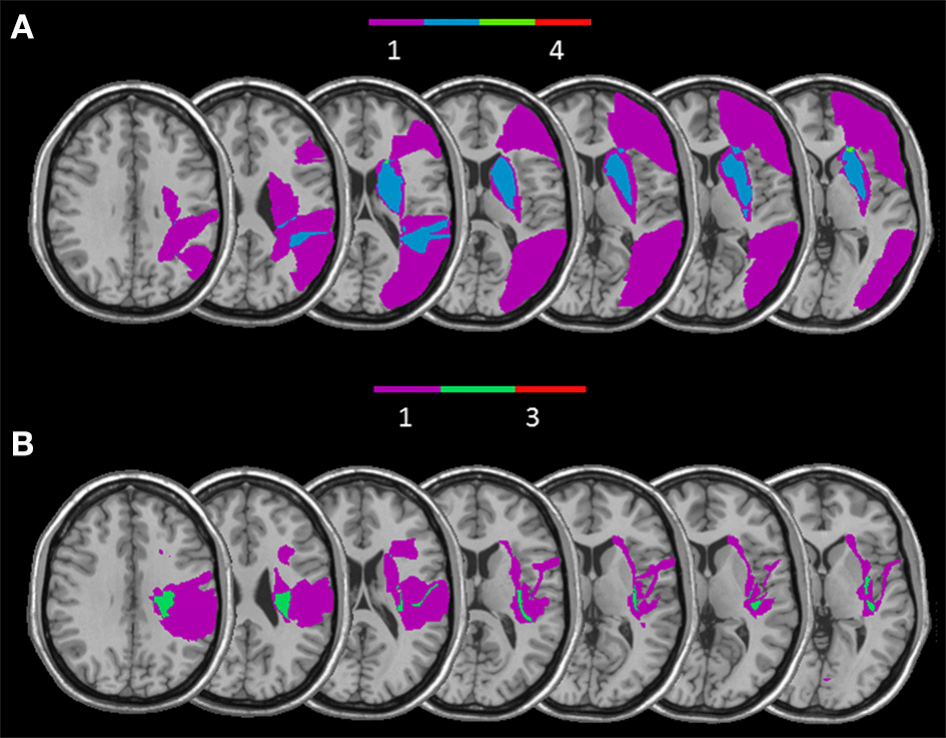
**Overlay of reconstructed lesion plots of RBD patients impaired with self-body processing in the Implicit (A) and in the Explicit task (B) superimposed onto MNI template**.

The RBD patients who showed a selective impairment in the Implicit task when self-body-parts was presented were affected by lesion involving a subcortical frontal region (caudate, putamen, internal, and external capsule and paraventricular area) and the temporal gyrus (for a graphical representation, see Figure 6A). Conversely, RBD patients selectively impaired in explicit self-body-parts recognition were affected by lesion involving the insular cortex and the cingulate gyrus (for a graphical representation, see Figure 6B). To exclude the dissociations observed within the RBD patients group were simply due to difference in the lesions extension, the Mann Whitney *U*-test (*U* = 4.00; *z* = 0.70*; p* = 0.50) was conducted to compare the average of voxels involved by the lesion. The lack of significance confirmed that the lesion extension did not differ across the two groups.

## Discussion

The main focus of the current study was to explore whether implicit and explicit recognition of self-body-parts could be selectively impaired after brain lesions. To this aim, thirty patients (15 RBD and 15 LBD) and thirty age-matched neurologically healthy subjects were submitted to two different tasks designed for testing implicit and explicit body-parts recognition.

In the Implicit task (Experiment 1), healthy subjects' performance was better when the displayed hand was their own hand compared to other people's hand, showing the so called self-advantage effect. In contrast, such self-advantage effect could no longer be observed in the Explicit task (Experiment 2). Indeed, when participants were required to judge if the displayed hand was their own hand, they were slower and less accurate with their own hand compared to others' hand.

Another critical difference between the Implicit and the Explicit task performance in healthy subjects is that different strategies were used to perform the two tasks. Mental motor rotation of body-parts is required to solve the laterality judgment task (Experiment 1; Parsons, 1987, 1994; Parsons and Fox, 1998; Ionta et al., 2007) while it is not necessary to explicitly recognize one's own hand (Experiment 2). The difference in task requirements is reflected in the classical bell-shaped function observed for response times only in Experiment 1 but not in Experiment 2 (see Figure 2). In order to perform the hand laterality judgment task (Implicit task) participants were required to recall the visuomotor representation of one's own body (Parsons, 1987, 1994). Thus, the self-advantage effect found in the Implicit task is likely to be closely related to the involvement of motor function. Taken together, data of Experiment 1 and Experiment 2 suggest that there are two ways to access to bodily self-knowledge: one way leading to an implicit, but not to an explicit knowledge, and the other way leading to an explicit knowledge.

Results on patients with a focal brain lesion confirmed the existence of these different ways. Indeed, different pattern of results were found in RBD and LBD patients. In the laterality judgment task, LBD patients were faster (and more accurate) when the displayed stimulus depicted their own hand compared with other peoples' hand, suggesting that they have implicitly recognized their own hand and a facilitation emerged in this condition. This facilitation for self-stimuli was not found in RBD patients. Moreover, when patients and controls' performance was compared only RBD patients, and not LBD patients, where selectively impaired in self-related stimuli.

As far as for the Explicit task, a worse performance in recognizing self than others' stimuli was found in controls as well as in brain damaged patients, independently from the left or right side of the lesion. Comparing patients' and healthy subjects' performance, again only patients with a right brain lesion were selectively impaired in recognize their own hand.

Since RBD patients, compared to the healthy subjects, were selectively impaired in processing of self but not others' body parts, and in agreement with our previous findings (Frassinetti et al., 2008), we speculate that, viewing a body-part and viewing one's own body-parts represent two functionally different processes involving distinct brain areas. Converging evidence have demonstrated that posterior regions, such as EBA and FBA, are activated en pictures of body and body-parts are presented (Downing and Peelen, 2016). On the other hand brain lesion mapping studies suggest that, in the right hemisphere, a fronto-parietal network as well as subcortical frontal regions are key for self-body processing (Frassinetti et al., 2008, 2009, 2012).

In addition to our previous knowledge, here behavioral and anatomical data put in evidence two, at least partially, distinct networks in the same right hemisphere involved in processing self-body stimuli when an explicit recognition is or it is not required. From a behavioral point of view, 40% RBD patients showed a selective deficit for implicit processing of self-body stimuli, whereas 27% of them showed a selective deficit in the explicit recognition of their own body. The overlap of RBD patients' lesions, who showed a selective impairment in implicit self-body-parts processing, mainly involved subcortical structures, such as the basal ganglia (caudate nucleus, putamen) and internal capsule, that are implicated in motor functions. Coherently, several neuroimaging studies showed that the ability to physically distinguish self from non-self-stimuli, such as one's own body from another's body and one's own action from another's action, primarily involved somatosensory and motor cortices (Uddin et al., 2005; Sugiura et al., 2006; Devue et al., 2007; Ferri et al., 2012). Further evidence derives from a recent study that applied single-pulse TMS to the right motor cortex and observed an increase in cortical excitability for self-specific stimuli when compared to non-self-specific stimuli (Salerno et al., 2012). Thus, both behavioral and anatomical data support the role of an integrated cortical–subcortical motor network in the right hemisphere in building the implicit knowledge of bodily self.

RBD patients selectively impaired in explicit self-body-parts recognition were affected by lesion involving the insular cortex and the cingulate gyrus. Noticeably, during the Explicit task participants had to give a judgment about the ownership of the observed hand. From this perspective, our results are consistent with earlier neuroimaging (Tsakiris et al., 2007) and neuropsychological (Karnath and Baier, 2010) studies showing that the right insula is involved in the explicit (or active) sense of body ownership (Tsakiris et al., 2010). Also, earlier studies suggested that both the insula and the cingulate cortex play a crucial role in the integration of body ownership and interoceptive awareness (Ehrsson, 2007). These studies used the rubber hand illusion, an experimental manipulation adopted to temporary altered the sense of body ownership. They demonstrated that threat to the rubber hand induce a correlation between the strength of the illusion and the cerebral activity evoked in the cingulate and insular cortices (Ehrsson, 2007).

Our findings are also in agreement with the dissociation between implicit and explicit forms of awareness in disorders concerning bodily recognition and sense of body ownership. Moro et al. (2011) investigated the neural correlates of implicit and emergent motor awareness in patients with anosognosia for hemiplegia. Analogous to our results here, they observed that deficits in implicit and emergent awareness are associated with damage to subcortical motor structures and insular regions, respectively (see also Moro, 2013). As far as the anosognosia for hemianestesia (AHE), in our sample, 2 out of 3 RBD patients affected by AHE showed a selective impairment in the explicit bodily self-recognition. Furthermore, one of them was also affected by corporeal neglect. We may suggest that we observed a co-occurrence of altered bodily self-awareness and altered ability to explicitly recognize the own one body. However, further studies will better clarify the relationship between the clinical deficit and the occurrence of bodily self-recognition impairment.

Patients with somatoparaphrenia and anosognosia do not show an explicit knowledge but can have spared implicit awareness of their body and of its motor potentialities (for a review see Vallar and Ronchi, 2006, 2009; Moro et al., 2008). However, to our knowledge, this is the first time that the opposite dissociation (an impaired implicit and a spared explicit knowledge) is described. This suggests, in line with our results in RBD patients, that within the self-body representation, the implicit and explicit knowledge, involved at least partially different brain regions, and thus may be selectively damaged following a brain lesion. Our patients did not show signs and did not report symptoms of somatoparaphrenia. Somatoparaphrenia has been reported, with a few exceptions, in right brain-damaged patients, with motor and somatosensory deficits, and it is most often characterized by a delusion of disownership of left-sided body parts (Vallar and Ronchi, 2009; Gandola et al., 2012). It is important to note that the conditions in which somatoparaphrenia emerges are very different from the experimental setting used in the present study. Indeed, here patients were asked to judge whether the pictures displayed on a computer screen depicted or not their own hand. By contrast, somatoparaphrenia is characterized by spontaneous limb disownership and confabulations concerning their affected limb. None of the RBD patients impaired in explicit self-hand recognition spontaneously confabulated about their affected limbs. Two, not alternative, hypothesis can be put forward in this respect. The first one is that the two deficits (somatoparaphrenia and the deficit here mentioned) refer to different body representations. The second one is that additional cognitive components are impaired in somatoparaphrenia. To verify this hypothesis a further study should be conducted comparing patients with and without somatoparaphrenia in the Implicit and Explicit task.

In sum, the present findings lead to consider that different brain lesions may cause specific deficits in bodily self-processing. Indeed, our results suggested the existence of two of distinct networks within the right hemisphere underlying implicit and explicit self-body recognition.

This could be particularly relevant for the diagnosis and rehabilitation of these disorders. Thus, the evaluation of implicit and explicit impairment in self-body processing should be included in the post-lesion neuropsychological assessment performed in the rehabilitative clinical practice. Furthermore, specific attention to the bodily self-processing should be carried out especially during the early phases following brain damage. Indeed, in these stages, plastic phenomena concerning both the brain and self-processing reorganization can occur. Thus, appropriate therapeutic strategies integrating sensorimotor, emotional and cognitive components may be introduced to support structure and functions of bodily reorganization of the self, including implicit aspects of the subjective experience.

## Author contributions

MC and FFr designed the study, analyzed the data and drafted the manuscript. MC performed data collection. FFe, MF, SA, GN, and VG critically revised the manuscript. All authors approved the final version of the manuscript.

## Funding

This work was supported by grants from RFO (Ministry of University and Research) and IRCSS Fondazione Maugeri (Italy) to FFr.

### Conflict of Interest Statement

The authors declare that the research was conducted in the absence of any commercial or financial relationships that could be construed as a potential conflict of interest.
