# Supplementary material for: Implicit and Explicit Routes to Recognize the Own Body: Evidence from Brain Damaged Patients
Source: Front Hum Neurosci. 2016 Aug 31;10:405. doi: 10.3389/fnhum.2016.00405 (PMC5006097; doi:10.3389/fnhum.2016.00405)
Supplement: Supplementary file 1 [file DataSheet1.DOCX]

Supplementary Material

Implicit and explicit routes to recognize the own body: evidence from brain damaged patients

# Michela Candini^1^, Marina Farinelli^2^, Francesca Ferri^3^, Stefano Avanzi^4^, Daniela Cevolani^5^, Vittorio Gallese^6^, Georg Northoff^7^, Francesca Frassinetti^* 1,4^

*** Correspondence:** Corresponding Author: [francesc.frassinetti@unibo.it](mailto:francesc.frassinetti@unibo.it)

*Table S1: Significance Test, point and interval estimates of the effect size for the differences between case and controls are individually reported for each patient.*

|  |  |  |  | **Significance Test** | | **Estimated Effect Size** |
| --- | --- | --- | --- | --- | --- | --- |
|  |  |  | **Accuracy** | **t** | **p** | **Point (95% CI)** |
| **Experiment 1** | | RBD 2 | 56% | -3,098 | p<.01 | -3,200 (-4,468 to -1,915) |
|  |  | RBD 3 | 58% | -2,905 | p<.01 | -3,000 (-4,200 to -1,781) |
|  |  | RBD 9 | 48% | -3,873 | p<.01 | -4,000 (-5,542 to -2,443) |
|  |  | RBD 10 | 61% | -2,614 | p<.03 | -2,700 (-3,801 to -1,579) |
|  |  | RBD 13 | 65% | -2,227 | p<.04 | -2,300 (-3,272 to -1,306) |
|  |  | RBD 15 | 44% | -4,26 | p<.01 | -4,400 (-6,081 to -2,705) |
| **Experiment 2** | | RBD 5 | 10% | -2,683 | p<.03 | -2,771 (-3,895 to -1,627) |
|  |  | RBD 6 | 16% | -2,441 | p<.03 | -2,521 (-3,564 to -1,457) |
|  |  | RBD 11 | 9% | 0,545 | p<.03 | 0,563 (0,007 to 1,100) |
|  |  | RBD 12 | 17% | -2,4 | p<.03 | -2,479 (-3,508 to -1,429) |

**Supplementary analysis:**

In order to check for the presence of biomechanical constraints effects in patients and controls, separate ANOVAs were conducted on RTs for the Implicit and Explicit Task with Posture (0° - 60° and 300° = Natural posture and 120° - 180° and 240° = Unnatural posture) as within-subjects factor and Group (H-R, H-L, RBD patients, LBD patients) as between-subjects factor.

Where necessary, post-hoc analyses were conducted by using Bonferroni’s correction. The magnitude of effect size was expressed by η^2^_p_.

**Experiment 1 (Implicit task):** The main effect of **Group** was significant [F(3,56)=6.61; p<.0001; η^2^_p_ =.26]: LBD patients responded slower than Controls (p<.01). The main effect of **Posture** was significant [F(1,56)=199.05 p<.0001; η^2^_p_ =.78] since RTs to stimuli oriented with unnatural posture (1749 ms) were longer compared to natural posture (1478 ms). The interaction Group x Posture was not significant.

**Experiment 2 (Explicit task):** The main effect of **Group** was significant [F(3,56)=11.9; p<.0001; η^2^_p_ =.39]: LBD and RBD patients responded slower than Controls (p<.003). The variable Posture was not significant [F(1,56)=1.51 p=.22]. The interaction Group x Posture was not significant.

Overall, these results further corroborate that participants adopted a motor strategy to solve the Implicit but not the Explicit task.

**
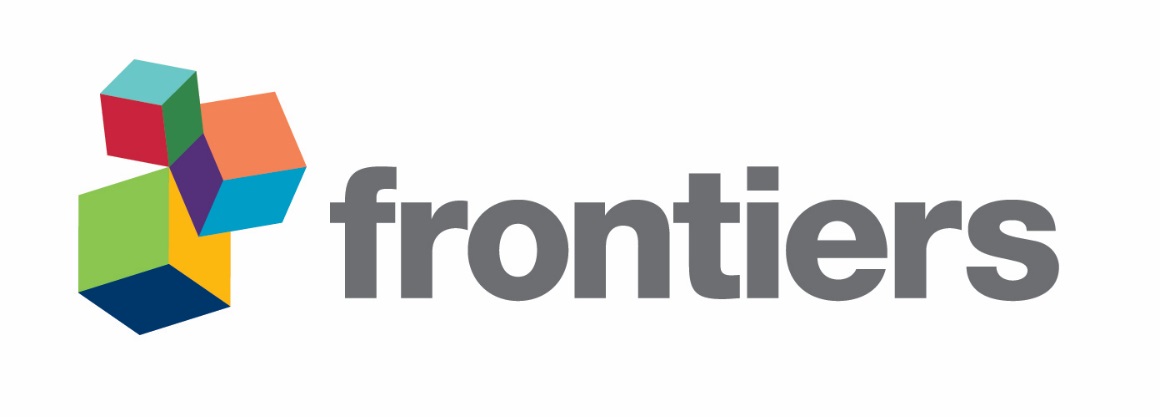
**

**Supplementary Figure 1.** The figure legends are required to have the same font as the main text, 12 point normal Times New Roman, single spaced. Please use a single paragraph for each legend and prepare the figures keeping in mind the PDF layout.
